# Antagonism of Na_v_ channels and α_1_-adrenergic receptors contributes to vascular smooth muscle effects of ranolazine

**DOI:** 10.1038/srep17969

**Published:** 2015-12-10

**Authors:** Anne Virsolvy, Charlotte Farah, Nolwenn Pertuit, Lingyan Kong, Alain Lacampagne, Cyril Reboul, Franck Aimond, Sylvain Richard

**Affiliations:** 1 PhyMedExp, INSERM U1046, UMR CNRS 9214, Université de Montpellier, Montpellier F-34295, France; 2 Avignon Université, LAPEC EA4278, F-84000, Avignon, France

## Abstract

Ranolazine is a recently developed drug used for the treatment of patients with chronic stable angina. It is a selective inhibitor of the persistent cardiac Na^+^ current (I_Na_), and is known to reduce the Na^+^-dependent Ca^2+^ overload that occurs in cardiomyocytes during ischemia. Vascular effects of ranolazine, such as vasorelaxation,have been reported and may involve multiple pathways. As voltage-gated Na^+^ channels (Na_v_) present in arteries play a role in contraction, we hypothesized that ranolazine could target these channels. We studied the effects of ranolazine *in vitro* on cultured aortic smooth muscle cells (SMC) and *ex vivo* on rat aortas in conditions known to specifically activate or promote I_Na_. We observed that in the presence of the Na_v_ channel agonist veratridine, ranolazine inhibited I_Na_ and intracellular Ca^2+^ calcium increase in SMC, and arterial vasoconstriction. In arterial SMC, ranolazine inhibited the activity of tetrodotoxin-sensitive voltage-gated Na_v_ channels and thus antagonized contraction promoted by low KCl depolarization. Furthermore, the vasorelaxant effects of ranolazine, also observed in human arteries and independent of the endothelium, involved antagonization of the α_1_-adrenergic receptor. Combined α_1_-adrenergic antagonization and inhibition of SMCs Na_v_ channels could be involved in the vascular effects of ranolazine.

## 

Ranolazine is a potent antianginal drug approved for the treatment of inadequately controlled chronic stable angina in adult patients ineligible for coronary revascularization and intolerant to first-line therapies (nitrates, β-blockers, Ca^2+^ antagonists). Clinical trials have shown that ranolazine reduces the symptoms of angina and improves exercise tolerance in patients with coronary heart disease^1^,^2^. Unlike conventional antianginal drugs that reduce heart rate or blood pressure, ranolazine acts on ventricular cardiomyocytes^3^,^4^. Reduction of electrical and mechanical dysfunction by ranolazine is thought to occur via the inhibition of the persistent Na^+^ current (I_Na_)^5^,^6^,^7^,^8^ that is enhanced during ischemia^9^. Through the preferential blockade of the persistent I_Na_, ranolazine prevents the Na^+^-induced Ca^2+^ overload that occurs during ischemia, ultimately protecting the myocardium and attenuating ischemia^10^,^11^. The electrophysiological consequences of ranolazine and its pharmacological effects on action potential duration and intracellular Na^+^ and Ca^2+^ homeostasis are critical for its therapeutic effects^12^.

Voltage-gated Na^+^ currents have been described in vascular smooth muscle cells (SMCs)^13^,^14^,^15^,^16^. In human coronary SMCs, I_Na_ has been recorded and has been shown to regulate intracellular Na^+^ and Ca^2+^ levels^13^,^17^. Vascular voltage-gated sodium channels (Na_v_) are sensitive to small changes in membrane potential and provide SMCs with an effective mechanism to elevate intracellular sodium [Na^+^]_i_, and, thereby, calcium [Ca^2+^]_i_ via the Na^+^-dependent activation of the reverse mode of the Na^+^/Ca^2+^exchanger (NCX)^18^,^19^. In rat arteries, it has been evidenced that Na_v_ channels contribute to the contractile response of SMCs^18^,^19^.

In addition to protecting the heart from the consequences of ischemia, recent evidence suggests that ranolazine also improves regional coronary blood flow and exerts a vasorelaxant effect comparable to that of nitroglycerin in magnitude, but more persistent^20^. Vasorelaxant responses to ranolazine have also been described in *ex vivo* and *in vivo* animal models, and could combine the blockade of α_1_-adrenergic receptors^21^,^22^,^23^ and voltage-gated Ca^2+^ channels antagonism (Ca_v_)^24^,^25^. However, the precise molecular mechanisms implicated have not been studied. It is unknown if Na_v_ channel inhibition could contribute to the vasorelaxant effect of ranolazine. Na_v_ channels are potential targets for ranolazine due to their role in regulating arterial contraction^18^,^19^. The present work aimed to explore the vascular effects of ranolazine and to elucidate the underlying molecular mechanisms.

## Results

### Effects of ranolazine on Na^+^ current in rat aortic SMCs

**Figure 1:**
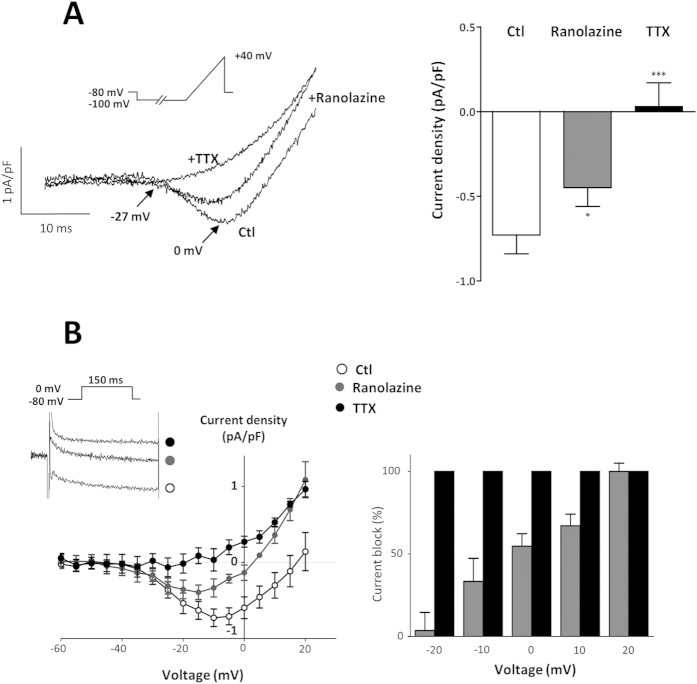
Ranolazine antagonizes veratridine-induced I_Na_ in rat aortic myocytes. (**A**) (*Left panel*) Representative I_Na_ current traces obtained on cultured SMCs in control (Ctl) and in the presence of 20 μM ranolazine (+ ranolazine) and 1 μM tetrodotoxin (+TTX). The current was revealed by a 40 ms ramp from −100 to +40 mV, following a 2 sec prepulse at −100 mV, from a holding potential of −80 mV in presence of veratridine (100 μM). Arrows indicate the activation and the maximal amplitude of the current with the corresponding voltages. (*Right panel*) Bar graph showing the averaged data expressed as mean ± sem (n = 10). (**B)** (*Left panel*) I_Na_ current-voltage relationships, obtained as described in A, for control (○, Ctl) and in the presence of ranolazine (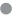) or TTX (●) (n = 10). The inset shows representative traces of the I_Na_ recorded for each condition at 0 mV from a HP of −80 mV. (*Right panel*) Bar graph showing I_Na_ block at various voltages, in the presence of ranolazine and TTX. Values expressed as percentage were calculated after subtraction of the TTX-insensitive current (n = 10). *p < 0.05, ***p <  0.001, one-way Anova followed by Bonferroni post-test.

I_Na_was evoked in rat aortic SMCs using either a voltage-ramp protocol or square depolarizations. In order to promote the current with sustained activation during depolarization, we used the Na_v_ agonist veratridine. In presence of veratridine (100 μM), I_Na_ activated at voltages positive to −30 mV and peaked around −10 mV (Fig. 1). We used the specific Na_v_ blocker tetrodotoxin (TTX) to validate that this current originated from Na_v_, and to quantify and specify the effect of ranolazine. In the presence of 1 μM TTX, all currents were blocked (Fig. 1A). Ranolazine (20 μM) blocked the TTX-inhibited I_Na_ at its maximal amplitude (Fig. 1A,B), reducing the current by 40%. In sharp contrast with the blocking effect of TTX, ranolazine inhibition of I_Na_ increased markedly with depolarization (Fig. 1B, right panel).

### Effects of ranolazine on intracellular Ca^2+^ in rat aortic myocytes

**Figure 2:**
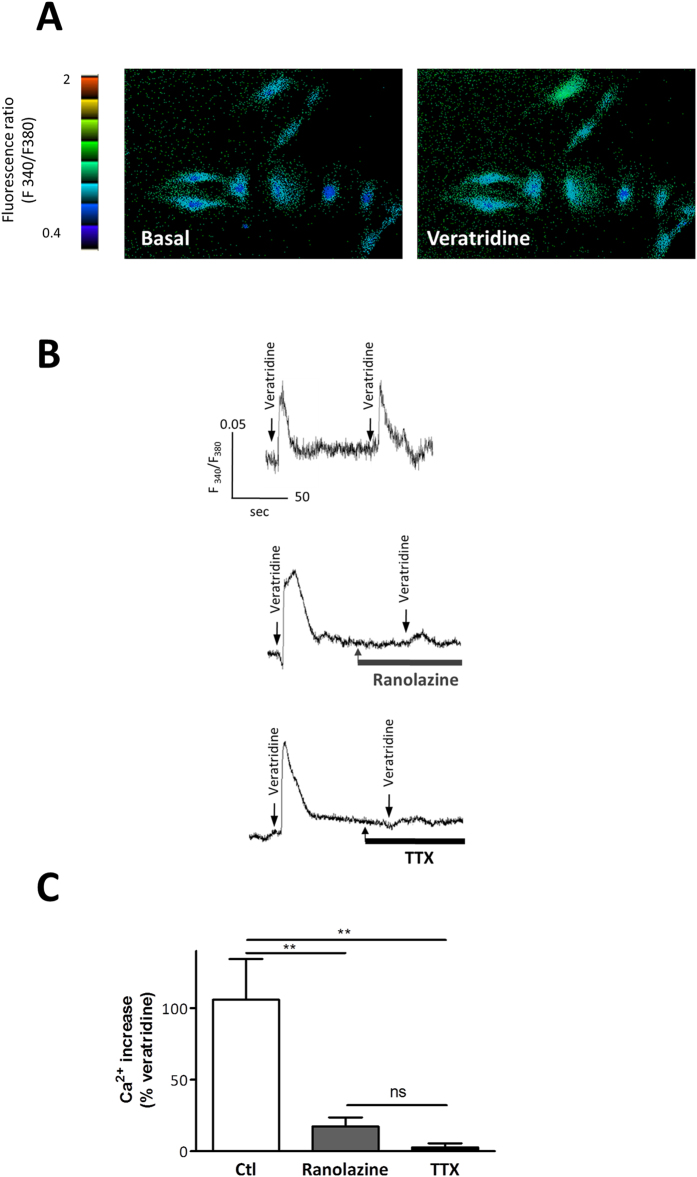
Ranolazine prevents the (Ca^+2^)_i_ increase induced by veratridine in rat aortic myocytes. (**A**) Pseudocolored images of the Fura-2 ratio (F340/F380) in cultured SMCs illustrating basal and veratridine-stimulated (Ca^+2^)_i_ levels. (**B**) Representative recordings of variations in the fluorescence ratio induced by veratridine (100 μM) in the absence or in the presence of ranolazine (20 μM) and TTX (1 μM). Arrows indicate the time of application of veratridine. (**C**) Bar graph representing the (Ca^+2^)_i_ increase induced by veratridine under various conditions. Changes in the fluorescence ratio induced by veratridine were determined under basal conditions and in the presence of ranolazine or TTX. Data are expressed as percent of the response induced by a first application of veratridine on the same cellular field and represent the mean ± sem of 6 different cell cultures (4 cover glasses/fields for each experimental condition per cell culture). **p < 0.01, Kruskal-Wallis one-way analysis of variance followed by Dunn’s test.

In primary cultured rat aortic SMCs, veratridine (100 μM) induced a transient and reproducible increase in [Ca^2+^]_i_ (Fig. 2). Ranolazine (20 μM) and TTX (1 μM) similarly inhibited the veratridine-induced [Ca^2+^]_i_ increase (Fig. 2). The veratridine response was completely blocked by TTX and was antagonized by 82.6 ± 6.2% by ranolazine. No antagonistic effect of either ranolazine or TTX was observed on the basal level of [Ca^2+^]_i_ suggesting that Na_v_ channels were not activated at rest.

### Ranolazine inhibited Na_v_ channel-dependent aortic contraction

**Figure 3:**
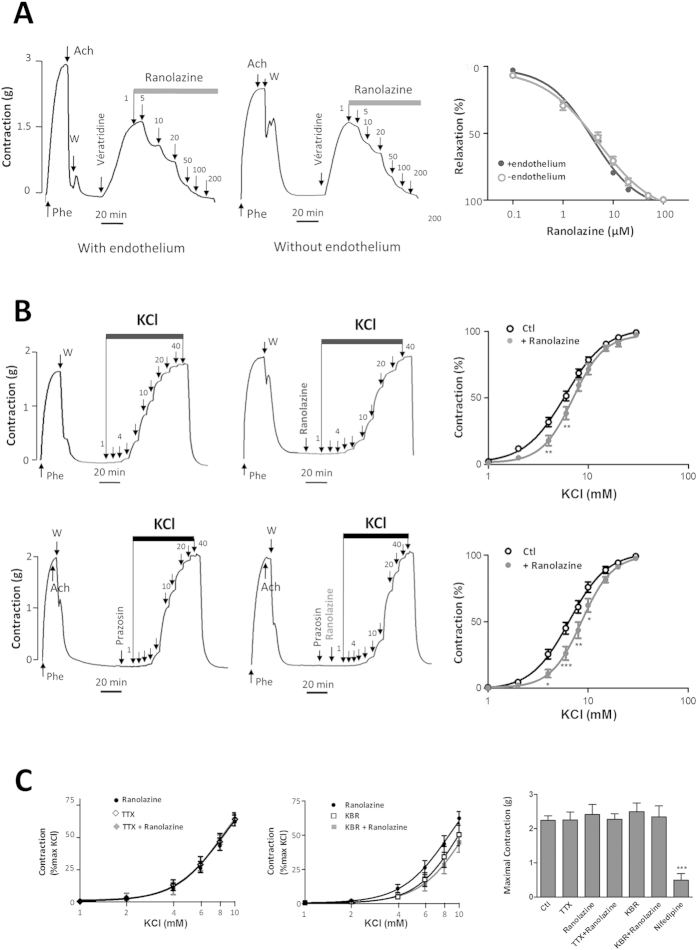
Implication of Na_v_ channels in the vascular response to ranolazine in rat aortic rings. (**A**) Ranolazine reversed the contraction induced by veratridine in the presence and in the absence of the endothelium. Typical recordings of variations in isometric tension during the following protocols were shown: the presence or absence of endothelium was first confirmed by either the induced vasorelaxation or the lack of an effect of 1 μM acetylcholine (Ach) on the contraction evoked by a submaximal concentration of Phe (10 μM), then after a wash period, ranolazine was cumulatively added (0.1 to 200 μM) after the contraction induced by veratridine (100 μM) was established. Graph summarizes dose-response curves to ranolazine (n = 10 aortas; each protocol performed in duplicate). (**B**) The effect of ranolazine was evaluated in the absence of endothelium on KCl-induced contraction under basal conditions (upper panels) and after α-adrenergic blockade with prazosin (lower panels). Typical recordings illustrate variations in isometric tension after the addition of cumulative doses of KCl (1 to 40 mM) in the absence (left) and in the presence of ranolazine (20 μM) (right). Graphs summarize the dose-response curves obtained for KCl. Data are expressed as the percentage of the maximal contraction induced by KCl (n = 15 aortas). The inset shows the maximal KCl-induced contraction (in g) for the control and in the presence of ranolazine and nifedipine (1 μM). (**C**) *(Left and middle panels)* The effects of ranolazine on KCl-induced contraction were evaluated in de-endothelialized aortic rings in the presence of prazosin, after inhibition of the Na_v_ with TTX (1 μM) or of the NCX with KB-R7943 (10 μM). Dose-response curves were compared for KCl concentrations below 10 mM in the absence and in the presence of ranolazine. *(Right panel)* Graph shows the maximal contractions (in g) induced, in the presence of prazosin (10 μM), by KCl for the control and in the presence of TTX, ranolazine, KBR or nifedipine (1 μM) (n = 6 aortas, each protocol performed in duplicate). *p < 0.05, **p < 0.01, ***p < 0.001, two-way Anova for dose responses and one-way Anova for maximal contractions followed by Bonferroni post-test.

In aortic rings, veratridine (100 μM) triggered an increase in tension corresponding to 44 ± 3% of the maximal contraction induced by phenylephrine (Phe, 10 μM) in the presence of endothelium and to 56 ± 2% without endothelium (Fig. 3A). The subsequent addition of ranolazine induced a dose-dependent relaxation at concentrations ranging from 0.1 to 100 μM, both in aortic rings with an intact endothelium (IC_50_ 2.5 ± 0.9 μM, n = 6) and in endothelium-free preparations (IC_50_ 2.9 ± 1.3 μM, n = 6) (Fig. 3A). Prior incubation with ranolazine (20 μM) abolished the contractile response to veratridine (not shown). These results showed that ranolazine prevents and reverses veratridine effects in an endothelium-independent manner and initiates vasorelaxation of the artery.

We next investigated the effects of ranolazine on the vascular smooth muscle contractility according to experimental protocols that we have previously designed to unmask Na_v_ channels contribution to contractile function^18^. Thereby, we compared responses to increasing concentrations of KCl by cumulative additions ranging between 2 and 40 mM in the absence or presence of ranolazine following or not α_1_-adrenergic receptor blockade with prazosin (10 μM). We observed that ranolazine (20 μM) prevented the contraction induced by low KCl concentrations (less than 10 mM and below EC_50_ value) (Fig. 3B) both in the absence and in the presence of prasozin. The inhibitory effect of ranolazine induced a rightward shift in the dose response curves with slight increases in the EC_50_ values: 7.5 ± 0.6 mM *vs.*6.1 ± 0.3 mM (p = 0.0316, *t*-test) in the absence of prazosin and 8.9 ± 0.7 mM *vs.* 7.1 ± 0.4 mM (p =  0.0349, *t*-test) in the presence of prazosin. Prazosin was also used in combination with a Na_v_ channels antagonist (TTX) to unmask the contribution of SMCs Na_v_ channels to the contraction induced by low KCl concentrations. In the presence of TTX (1 μM), the KCl response was rightward shifted for concentrations below 10 mM, reflecting Na_v_ channel inhibition. The same effect was obtained with ranolazine (20 μM). There was no additional inhibition of ranolazine in the presence of TTX (Fig. 3C). The same inhibitory profiles were obtained with KB-R7943 (10 μM), a blocker of the reverse mode of the NCX^26^. We observed no difference between contractile responses to low KCl concentrations either in presence of ranolazine, KB-R7943 or KB-R7943 plus ranolazine (Fig. 3C). Ranolazine had no additional effect after NCX blockade. In Fig. 3C, the bar graph demonstrates that the maximal contractile response to 80 mM KCl either in presence of TTX, ranolazine or KBR was unchanged while a robust inhibition was observed in presence of nifedipine (1μM), a Ca^2+^ channel blocker.

### Ranolazine inhibited α_1_-adrenergic-dependent rat aortic contraction

**Figure 4:**
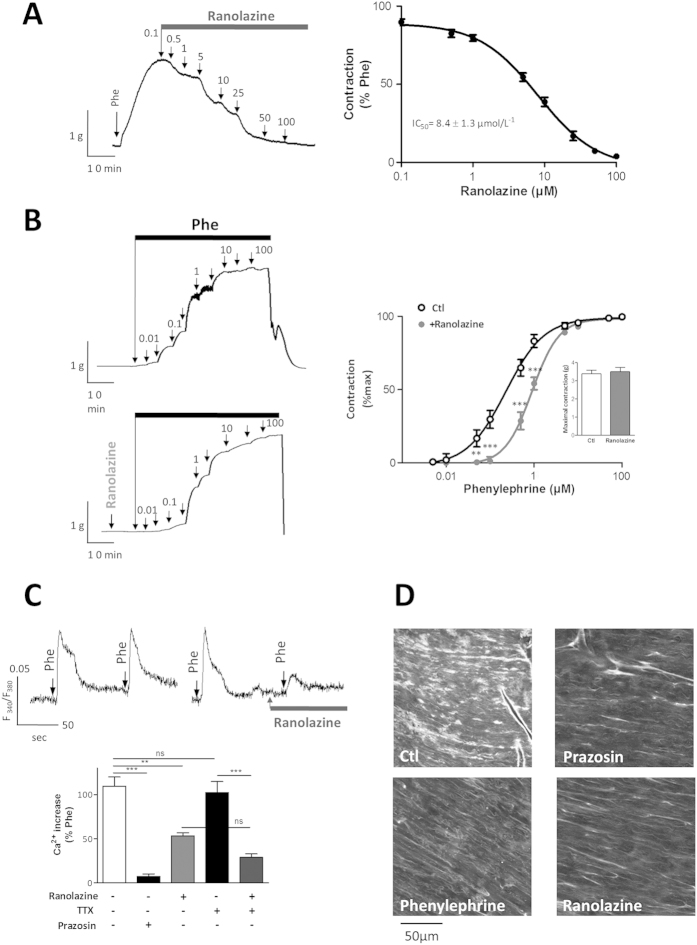
Ranolazine antagonizes the α-adrenergic response. (**A**) The effect of ranolazine was evaluated on the contraction induced by a submaximal concentration of Phe. The left panel illustrates typical relaxation induced by cumulative concentrations of ranolazine (0.1 to 200 μM) when the aorta was previously contracted with Phe (1 μM). Right panel shows dose-response curve for ranolazine. Data represent the percentage of contraction relative to the maximal tension induced by Phe (n = 10 aortas, each protocol performed in duplicate). (**B**) The contractile response to Phe was evaluated in the absence or in the presence of ranolazine. Left panels illustrate variations in isometric tension induced by cumulative concentrations of Phe under basal conditions (top) and after a 15-min incubation with ranolazine (20 μM; bottom). Graph shows dose-response curves for Phe under each condition (n = 10 aortas, protocol performed in duplicate). The inset shows the maximal contraction (in g) induced by Phe. (**C**) Effect of ranolazine on the Phe-induced (Ca^+2^)_i_ increase on cultured SMCs. (*Upper panel*) Representative recordings of the fluorescence ratio illustrate the increase induced by Phe (1 μM) in the absence or in the presence of ranolazine (20 μM) on cultured SMCs. Arrows indicate the time of application of Phe. (*Lower panel*) Bar graph representing the (Ca^+2^)_i_ increase induced by Phe for basal condition (Ctl) and in the presence of prazosin (10 μM), ranolazine (20 μM), TTX (1 μM) or TTX plus ranolazine. Data are expressed as percent of the response induced by the first application of Phe on the same cellular field and represent the mean ± sem of 5 different cell cultures (average of 4 cover glasses/fields for each experimental condition per cell culture). (**D**) Effect of ranolazine on the binding of fluorescent prazosin (QABP) in the rat aorta. The control (Ctl) shows the intensity of fluorescence obtained with QABP alone. Non-fluorescent antagonists (prazosin, Phe and ranolazine) were used to compete with QABP for binding, resulting in reduced fluorescence. **p < 0.01; ***p < 0.001, two-way Anova followed by Bonferroni post-test for vascular reactivity and Kruskal-Wallis one-way analysis of variance followed by Dunn’s test for Ca^2+^ imaging.

Since antagonistic effects of ranolazine on the α_1_-adrenergic receptor have been reported, we investigated if this pathway is involved in the effects of ranolazine on arterial contraction in our model. We observed that ranolazine induced a dose-dependent relaxation (IC_50_  8.4 ± 1.3 μM; n = 6) of rat aorta previously contracted with a non-maximally active concentration of Phe (1 μM) (Fig. 4A). In the presence of ranolazine (20 μM), the dose-dependent response to Phe was shifted to the right (Fig. 4B), consistent with a competitive inhibition that was likewise correlated to ranolazine concentration (not shown). Furthermore, no effect of ranolazine was observed on the maximal response to Phe (Fig. 4B-inset).

The competitive antagonization of the α_1_-adrenergic receptor with ranolazine was confirmed on [Ca^2+^]_i_ levels in cultured SMCs (Fig. 4C) and on the binding of a α_1_-adrenergic agonist *in situ* on rat aortic SMCs (Fig. 4D). We observed that Phe induced a transient and reproducible increase in [Ca^2+^]_i_ (Fig. 4C). This response was antagonized by ranolazine (20 μM), suppressed by the positive control prazosin (10 μM) and insensitive to TTX (1 μM) both in absence and presence of ranolazine (Fig. 4C). Prazosin binds the α_1_-adrenergic receptor, as illustrated by the fluorescent signal reflecting BODIPY FL-Prazosin binding at the SMCs level and widely distributed through the media (Fig. 4D, CTL). This fluorescence signal was strongly reduced in the presence of ranolazine (Fig. 4D, ranolazine) as well as in the presence of non-fluorescent control antagonists (Fig. 4D, prazosin and Phe).

### Effect of ranolazine on human uterine arteries

**Figure 5:**
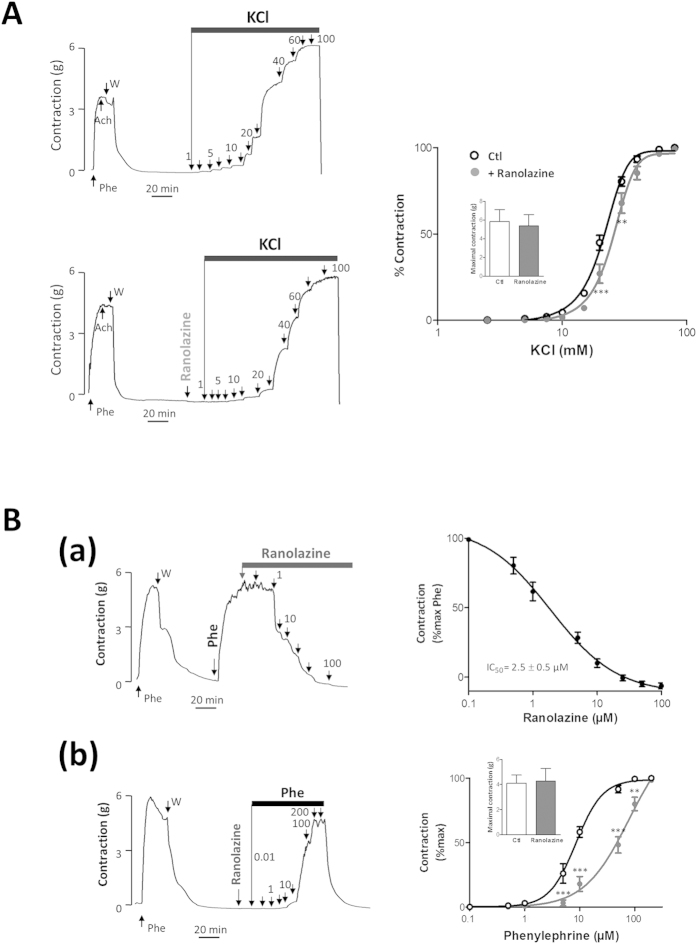
Vasorelaxant effects of ranolazine on human uterine arteries. Ranolazine inhibition of Na_v_ channels and α-adrenergic responses was observed in human uterine arteries. (**A**) The effects of ranolazine were evaluated in the absence of endothelium on the contractile response to KCl. Typical recordings illustrate the variations of isometric tension induced by cumulative addition of KCl (1 to 80 mM) in the absence and in the presence of ranolazine (20 μM). The graph shows dose-response curves for KCl under basal condition (Ctl) and in the presence of ranolazine. Data represent the percentage of contraction relative to the maximal tension induced by KCl. The inset shows the maximal contraction (in g) induced by KCl for the control and in the presence of ranolazine. (**B**) The effects of ranolazine were evaluated on the contractile response of uterine artery to Phe. (a) Arterial segments previously contracted with a submaximal concentration of Phe (10 μM) were then subjected to vasorelaxation induced by cumulative concentrations of ranolazine (0.1 to 100 μM). The right panel shows the dose-response curve for ranolazine. Data represent the percentage of contraction relative to the maximal tension induced by Phe (10 μM). (b) The contractile response to Phe was evaluated in the presence of ranolazine (20 μM) and the dose-response curve was compared to that obtained in absence of ranolazine. The inset shows the maximal contraction (in g) induced by Phe (200 μM) for the control and in the presence of ranolazine. Data were obtained from 6 different specimens of uterine arteries; each protocol was performed in triplicate. **p < 0.01, ***p < 0.001, two-way Anova followed by Bonferroni post-test.

To investigate the potential therapeutic relevance of our results, we performed experiments in human arteries (Fig. 5). In human uterine artery, ranolazine (20 μM) prevented the contractile response to low KCl concentrations (less than 30 mM and below EC_50_ value) similarly to that seen on rat aorta (Fig. 5A). In the presence of ranolazine, the dose response curve of KCl was rightward shifted and the EC_50_ value was increased (21.4 ± 0.8 m M*vs.* 26 ± 1.7 mM, p = 0.0127, *t*-test). No inhibitory effect of ranolazine was observed on the maximal contractile response to KCl (Fig. 5A-inset). This effect reflected, at least partially, inhibition of Na_v_. Additionally, ranolazine induced a vasorelaxation of human uterine arteries contracted after application of a non-saturating concentration of Phe (10 μM) (Fig. 5B-a). The effect of ranolazine was dose-dependent with an IC_50_ value of 2.5 ± 0.5 μM consistent with therapeutic concentrations. In the presence of ranolazine (20 μM), the dose-dependent response to Phe was significantly shifted to the right, reflecting competitive inhibition of the α_1_-adrenergic receptor (Fig. 5B-b) whereas no effect was observed on the maximal contractile response to Phe (Fig. 5B-b-inset).

## Discussion

The antianginal properties of ranolazine have been attributed primarily to the inhibition of the persistent I_Na_ in cardiomyocytes^5^,^6^,^7^,^8^,^27^. In the present study, we show that the vasorelaxant effect of ranolazine in arteries involves antagonism of α_1_-adrenergic receptors and inhibition of Na_v_ channels at the smooth muscle level.

**Figure 6:**
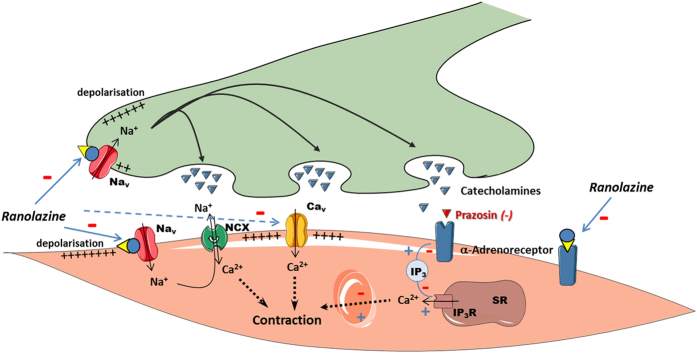
Representation of the vascular effects of ranolazine involving Na_v_ channel inhibition in vascular myocytes and in sympathetic nerve endings, and α_1_-adrenergic receptor antagonization. Na_v_: voltage-gated sodium channels; Ca_v_: voltage-gated calcium channels, NCX: sodium-calcium exchanger; IP_3_R: IP_3_ receptor; SR: sarcoplasmic reticulum. The dotted arrow illustrates Ca_v_ channel antagonism as reported previously by Deng *et al.*^24^ and Malavaki *et al.*^25^

One major finding of our study is that Na_v_ channels, present in arteries, are possible targets of ranolazine and could participate in the vasorelaxant effects of the drug. Previously, we had evidenced a TTX-sensitive component of tension in the rat aorta which is comprised of two mechanisms^18^ (Fig. 6). One mechanism involves Na_v_ channels isoforms from the vascular myocytes. Na^+^ entry through the SMCs Na_v_ channels triggers Ca^2+^ influx through the reverse mode of the NCX and, thereby, promotes contraction^17^,^18^,^19^. The other mechanism involves the activity of Na_v_ channels at sympathetic perivascular nerve terminals and impacts catecholamine release with subsequent α_1_-adrenergic receptor activation. Both mechanisms were potentially inhibited by ranolazine.

We have shown an inhibitory effect of ranolazine on SMC Na_v_ channels, both directly on a persistent I_Na_ (Fig. 1) and indirectly by prevention or abolition of the intracellular Ca^2+^ rise (Fig. 2) and contraction (Fig. 3) promoted by the alkaloid Na_v_ agonist veratridine. Veratridine prevents the inactivation and deactivation of the Na_v_ channel, thereby promoting persistent Na^+^ influx and consequently a rise in [Ca^2+^]_i_ via a cascade of pathways which elicits contraction^18^,^19^ and involves the NCX reverse mode^13^,^28^, Ca^2+^-activated Cl^-^ channels and voltage-activated Ca^2+^ channels^29^. The effects of ranolazine on Na^+^ influx and Ca^2+^ homeostasis evidenced here in vascular myocytes are very similar to those described in cardiac myocytes^27^. Mechanistically, the use of an agonist (veratridine) or of a weak depolarization following addition of low KCl concentration was required to unravel ranolazine activity on the SMCs Na_v_ channels. This is in line with previous findings that Na_v_ channels need to be activated prior to seeing an effect of the drug and consistent with an open-state blocking mechanism^30^. The effect of ranolazine was steeply voltage-dependent with inhibition being enhanced by increasing depolarization promoting channel opening. This mechanism corresponded to the electrophysiological properties of the drug previously demonstrated in cardiac myocytes^31^. This contrasted markedly with that of TTX whose mechanism of action is to form a plug in the pore of the channel independently of voltage. In addition to vascular SMCs Na_v_ channels, ranolazine may also target Na_v_ channels at sympathetic perivascular nerve terminals. Although it is complex to specifically study Na_v_ channels at the sympathetic perivascular nerve terminals, we could speculate that the inhibitory effect of ranolazine also affects these Na_v_channels when activated.

Voltage-gated Ca^2+^ (Ca_v_) channel inhibition has also been reported in the vascular effects of ranolazine^24^,^25^. In our study, such antagonization should be considered, especially as Ca_v_ channels are also implicated in veratridine-induced events^17^. However, we observed that ranolazine did not mimic the effect of the Ca_v_ channel antagonist nifedipine. Nifedipine inhibited KCl-induced contraction, particularly in the maximal contractile response. High concentrations of KCl strongly depolarize cells and Ca_v_ channels are predominantly involved in the resulting contractile response. Absence of an inhibitory effect on that response revealed no antagonism on these channels as is the case for TTX and KBR. We observed that ranolazine, at a concentration in line with therapeutic doses (20 μM), did not affect the response to high KCl concentration. In our conditions, Ca_v_ channels inhibition was not substantially involved in the vasorelaxant effects of ranolazine.

Another mechanism implicated in the vasorelaxant effects of ranolazine could potentially be the antagonization of α_1_-adrenergic receptor^22^,^24^,^25^,^32^. Indeed, we demonstrated an inhibitory effect of ranolazine at concentrations corresponding to therapeutic doses, on both arterial contraction and intracellular rise of Ca^2+^ in aortic SMCs induced by Phe. The stimulation of α_1_-adrenergic receptors regulates arterial blood pressure in the rat aorta^33^,^34^ and modulates vasoconstriction in coronary arteries. There is little α_1_-adrenergic coronary vasomotor tone at rest but α_1_-adrenergic hyperactivity can be promoted by atherosclerosis and thereby can contribute to myocardial ischemia^29^,^35^,^36^. Consistently, we observed no effect of either ranolazine or prazosin on vascular tone at rest, in line with the absence of α_1_-adrenergic tone at rest, and vasorelaxation was achieved only when the α_1_-adrenergic system was stimulated.

Ranolazine has multiple molecular targets and is not highly specific^9^,^37^ but it is thought to reduce electrical and mechanical cardiac dysfunctions by inhibition of persistent I_Na_ in cardiomyocytes^27^,^38^. The current view of the therapeutic benefits of ranolazine in stable ischemic angina is that they arise from the normalization of cardiac Na_v_ channel activity and, consequently, of Na^+^ and Ca^2+^ overload in ischemic cardiomyocytes^39^. Improvement of regional coronary perfusion was also suggested but no molecular mechanism has been proposed^20^.

Our results are consistent with the idea that vasorelaxant properties of ranolazine may improve myocardial perfusion under ischemic conditions. Although we had no access to human coronary arteries to assess the effect of ranolazine on their contractile activity, previous identification of Na_v_ channels involved in intracellular Na^+^ and Ca^2+^ overload in coronary SMCs is consistent with this hypothesis^13^,^17^. These channels represent a contractile reserve that could significantly impact vascular tone especially in resistance arteries^19^. Although several studies have reported the functional coupling between Na_v_ channels and arterial contraction, no pathophysiological situation involving that regulation has been clearly identified. However, it has been shown that hypoxia can induce vasoconstriction which is sensitive to Na_v_ channels blockers^40^. Hypoxic conditions mimic pathological situations such as angina; ranolazine through vascular Na_v_ channels inhibition could regulate vascular tone in these circumstances.

The α_1_-adrenergic receptors are also critical to vasoconstriction in human coronary arteries, and are involved in enhanced vasoconstriction at both the epicardial and microcirculatory levels in atherosclerotic conditions^41^. This also further strengthens our rationale and working hypothesis for potential therapeutic benefits of ranolazine at the coronary level under ischemic conditions or following different types of coronary manipulation and intervention (for review see^36^). We hypothesize that dynamic coronary stenosis could be reversed by ranolazine through an antagonistic action on the α_1_-adrenergic mediated vasoconstriction^42^.

Clinical trials have reported a possible association of anti-anginal properties of ranolazine and improvement of regional coronary blood flow^20^,^43^,^44^. However, ranolazine is presented as devoid of hemodynamic effects^45^,^46^ whereas α_1_-adrenergic receptor antagonists used to treat hypertension have side effects such as orthostatic hypotension or tachycardia^47^. Interestingly, a few events of orthostatic hypotension in healthy volunteers have been reported with high doses of ranolazine (2000 mg) while no such side effect was observed at therapeutic doses (500–1000 mg)^3^. The IC_50_ values that we determined for both Na_v_ channels and α_1_-adrenergic receptors are in the range of therapeutic concentrations. At these concentrations, ranolazine induced a partial vasorelaxation and exhibited no vasodilatory effect. At higher concentrations vasorelaxation is pronounced and almost complete. This could explain the absence of hemodynamic effects and is in line with clinical observations.

## Conclusion

Although the inhibition of the persistent I_Na_ has been well-established in cardiomyocytes as the mechanism responsible for ranolazine’s antianginal properties, the inhibition of persistent Na^+^ influx through arterial Na_v_ channels, together with an antagonization of α_1_-adrenergic system over activation, may also contribute significantly to its therapeutic action. Pharmacologically, ranolazine inhibits the activity of voltage-gated Na_v_ channels both at the level of aortic myocytes and, potentially, at sympathetic perivascular nerve terminals thereby inhibiting catecholamine release in addition to inhibiting α_1_-adrenergic receptors which seems relevant for the antianginal effects of the drug (Fig. 6). Therefore, the therapeutic effects of ranolazine may comprise both “upstream” benefits, by preventing or stopping vasoconstriction, and down-stream therapy involving the normalization of Na^+^ and Ca^2+^ overload in cardiomyocytes.

## Methods

### Preparation of vascular tissue and myocytes

Investigations on animal tissue conformed to the guidelines for the Care and Use of Laboratory Animals (NIH, N°.85–23, revised 1996) and European directives (2010/63/EU)) and were approved by the committee for Animal Care of Montpellier-Languedoc-Roussillon (N° CEEA-LR-12075). Experiments were performed on male Sprague-Dawley rats (22–25 weeks) anesthetized with an intraperitoneal injection of pentobarbital (60 mg/kg). The human tissues used in this study were considered as surgical waste in accordance with French ethics laws (L.1211-3 – L.1211-9), and their use was approved by the national ethics Committee and the French Ministry of Research (DC-2008-488). Specimens of uterine arteries were obtained after written consent from non-pregnant women (aged 40–60 years) undergoing hysterectomy for benign gynaecological disorders.

Arterial tissues (rat thoracic aorta and human uterine arteries) were immersed in a physiological saline solution (PSS, in mM: 140 NaCl, 5 KCl, 1 MgCl_2_, 0.5 KH_2_PO_4_, 0.5 Na_2_HPO_4_, 2.5 CaCl_2_, 10 HEPES and 10 glucose, pH 7.4), cleaned of fat and connective tissue, and cut into 2–3 mm-wide rings. When required by the experiment, the endothelium was removed by rubbing.

Isolated myocytes were obtained from the rat aorta by enzymatic dispersion of the media layer after mechanical removal of the adventitia. The tissue was incubated for 20 min at 37 °C in sterile PSS containing collagenase (1 mg/ml) and elastase (50 UI/ml). Cells harvested after mechanical dissociation were filtered through a nylon mesh,centrifuged at 250 g for 5 min and then seeded onto collagen-treated Petri dishes and cultured in specific smooth muscle growth medium (PromoCell, Heidelberg, Germany). Smooth muscle cells (SMCs) were sub-cultured once they reached 80%–90% confluence and were used between passages 2 and 5.

### Electrophysiological recordings

Cellular electrophysiological recordings were performed, at room temperature (22–24 °C), on cultured arterial SMC under the whole-cell patch clamp configuration. Experiments were conducted using an Axopatch 200B amplifier (Axon Instruments), interfaced to a Dell microcomputer with a Digidata 1440A Series analog/digital interface (Axon), using pClamp 10 (Axon). Recording pipettes were filled with (in mM): 120 CsCl, 5 MgCl_2_, 11 EGTA, 10 HEPES, 1 CaCl_2_, 5 ATP-Na_2_ and 10 TEA-Cl (pH 7.3 with CsOH). The bath solution contained (in mM): 135 NaCl, 1 CaCl_2_, 1 MgCl_2_, 10 HEPES, 10 glucose, 2 NiCl_2_(pH 7.4 with CsOH) and 0.1 veratridine. Our experimental conditions were optimized to record only voltage activated I_Na_. We used NiCl_2_ (2 mM in bath solution) to block Ca_v_ channels^48^. In addition, CsCl (120 mM, instead of KCl in the recording pipette) was used to inhibit K^+^ currents. Veratridine (100 µM) was added to promote sustained I_Na_ inactivation. Whole-cell membrane capacitances and series resistances were compensated electronically prior to recording. Voltage errors resulting from the uncompensated series resistance were always ≤8 mV and were not corrected. Experimental data were filtered on-line at 10 kHz prior to digitization and storage. The presence of I_Na_ current was revealed by the use of a ramp protocol defined as followed: from a holding potential (HP) of −80 mV, a −100 mV prepulse was applied for 2 sec, followed by a voltage ramp from −100 to +40 mV for 40 ms. Current/voltage I_Na_ relationship was obtained in response to 150 ms voltage steps to potentials between −60 to +20 mV from a HP of −80 mV; voltage steps were applied in 5 mV increments at 1 s intervals.

### Measurement of intracellular Ca^2+^ variations

Intracellular Ca^2+^ variations ([Ca^2+^]_i_) in cultured SMCs were measured using the ratiometric fluorescent Ca^2+^ indicator Fura-2 as previously described^49^,^50^. SMCs sub-cultured for 4 days in Lab-Tek II® chambers (Nunc, USA) were loaded with 2.5 μM Fura-2AM plus 0.02% Pluronic F-127. Cells rinsed with PSS were maintained in basal buffer during a 15-min waiting period for the de-esterification of Fura-2AM and chambers were mounted on a microscope stage (Axiovert, Zeiss, Germany; 20x objective). Buffer and drugs were then applied by perfusion to the cells as indicated in the figure legends. Cells were illuminated by excitation with a dual UV light source at 340 nm and 380 nm using a lambda DG-4 excitation system (Sutter Instrument Company, CA, USA). Images were captured digitally every 0.35 seconds with a cooled CCD camera (Photometrics, Roper scientific, France) at 510 nm emission. Changes in [Ca^2+^]_i_ were deduced from variations in the F340/F380 ratio after correction for background and dark currents (Metafluor software, Universal Imaging Corporation, USA). Data were averaged (at least 25 cells per field chosen randomly; one field per cover glass; 4 cover glasses for each experimental condition), with n representing the number of cell cultures.

### Isometric tension recording

Arterial segments were mounted between two stainless steel hooks placed in a conventional vertical organ bath chamber filled with 5 ml of PSS, maintained at 37 °C and continuously bubbled with O_2_. Changes in isometric tension were measured as previously described^18^ using an IT1-25 force transducer and an IOX computerized system (EMKA Technologies, France). Each arterial segment was subjected to a 60-min equilibration period at a basal resting tension of 2 g and its contractile function was assessed with 1 μM phenylephrine (Phe). In some experiments, the successful removal of the endothelium was confirmed by the inability of acetylcholine (Ach, 1 μM) to induce relaxation in Phe-contracted rings. After washout and a 20–30 min period of stabilization, protocols were followed as detailed in the legends. Concentration-response curves were generated by cumulative increases in the concentration of various agents: Phe, the depolarizing agent KCl and ranolazine. For specific protocols, prazosin (10 μM), tetrodotoxin (1 μM, TTX), KB-R7943 (10 μM, KBR) and nifedipine (1 μM) were used to block α_1_-adrenergic receptors, Na_v_ channels, the reverse mode of NCX and Ca_v_ channels, respectively. Rings were incubated with each compound for a 15-min period before dose responses were generated. KCl was added, at the indicated concentrations, to basal PSS containing 5.5 mM K^+^. Each experimental protocol was performed in duplicate (rat aorta) or triplicate (uterine artery), with n representing the number of individual.

### Fluorescent ligand binding to α_1_-adrenergic receptors

Segments of rat aorta were sliced open, cleared of adventitia and incubated in the dark for one hour at room temperature with BODIPY FL-Prazosin (QAPB, 100 nM), as previously described by others^51^. Once QABP binding equilibrium was reached, the following non-fluorescent antagonists were added to the incubation media for one hour at saturating concentrations to compete for QABP binding sites in segments from the same aorta: prazosin (10 μM), Phe (10 μM) and ranolazine (100 μM). Arterial segments were observed with a 40x oil-immersion objective, on an inverted Zeiss LSM Exciter laser scanning microscope (Zeiss, LePecq France). Optical images were collected at an excitation/emission of 488/515 nm for QAPB. Laser intensity, gain and offset (contrast and brightness) were kept constant for each artery and acquisition. Tissue was scanned at 1 μm intervals from the internal elastic lamina through the media, yielding z-series in stacks of approximately 20–50 μm in depth. Each condition was tested in triplicate on five different aortas.

### Chemical reagents

TTX and KB-R7943 were obtained from Tocris Biosciences (UK) and culture medium from PromoCell (Germany). All other chemicals and compounds were purchased from Sigma-Aldrich (France). KB-R7943 was dissolved in DMSO, veratridine in 0.1N HCl and the remaining compounds in distilled water with further dilutions made from stock solutions with PSS.

### Data analysis

All data are expressed as means ± standard errors of the mean (SEM) with the number of experiments indicated as *n*. Data were analyzed using GraphPad software (USA). Statistics were performed using either the Student’s *t*-test or two-way analysis of variance followed by Bonferroni post-test for two-group comparison or Kruskal-Wallis one-way analysis of variance followed by Dunn’s test for multiple-groups comparison. P values lower than 0.05 were considered significant.

## Additional Information

**How to cite this article**: Virsolvy, A. *et al.* Antagonism of Na_v_ channels and a_1_-adrenergic receptors contributes to vascular smooth muscle effects of ranolazine. *Sci. Rep.* **5**, 17969; doi: 10.1038/srep17969 (2015).
